# Bone biomarker for the clinical assessment of osteoporosis: recent developments and future perspectives

**DOI:** 10.1186/s40364-017-0097-4

**Published:** 2017-05-18

**Authors:** Tsung-Rong Kuo, Chih-Hwa Chen

**Affiliations:** 1 Graduate Institute of Nanomedicine and Medical Engineering, College of Biomedical Engineering, Taipei Medical University, Taipei, 11031 Taiwan; 2 International Ph.D. Program in Biomedical Engineering, College of Biomedical Engineering, Taipei Medical University, Taipei, 11031 Taiwan; 3 Bone and Joint Research Center, Department of Orthopedics and Traumatology, Taipei Medical University Hospital, Taipei, 11031 Taiwan; 4 School of Medicine, College of Medicine, Taipei Medical University, Taipei, 11031 Taiwan

**Keywords:** Bone biomarker, Bone formation, Bone resorption, Regulators, Bone turnover, Osteoporosis

## Abstract

Bone biomarkers included formation, resorption and regulator are released during the bone remodeling processes. These bone biomarkers have attracted much attention in the clinical assessment of osteoporosis treatment in the past decade. Combination with the measurement of bone mineral density, the clinical applications of bone biomarkers have provided comprehensive information for diagnosis of osteoporosis. However, the analytical approaches of the bone biomarkers are still the challenge for further clinical trials. In this mini-review, we have introduced the functions of bone biomarkers and then recently developed techniques for bone biomarker measurements have been systematically integrated to discuss the possibility for osteoporosis assessment in the early stage.

## Background

Osteoporosis is a worldwide disease with reduction of bone mass and decrease of bone strength to result in bone fragility and fracture. Based on the report of World Health Organization (WHO), the disease of osteoporosis has been diagnosed by bone mineral density (BMD) at the hip and/or the spine at least 2.5 standard deviations below in comparison with the bone mass of young healthy adults as determined by dual-energy X-ray absorptiometry (DXA) [1]. The people with osteoporosis are steadily increased because of aging society occurring worldwide. There are about 200 million people are suffered from osteoporosis in the word and approximately 8.9 million fractures are caused by osteoporotic fracture [2]. In the osteoporotic fractures, hip fractures have led to mortality rates up to 20–24% within the first year and then the death rate has steadily increased for at least 5 years [3]. After hip fractures, the survivors may lose the capability of action and independence with 40% unable to walk independently and 60% requiring assistance at least 1 year. Due to the loss of capability, around 33% patients are totally dependent or in a nursing home in the year following a hip fracture. Nowadays, osteoporosis is a major concern of public health because of its healthcare cost. Moreover, the fracture caused by osteoporosis is the most important factor for the decreases of quality of life and survival rate in aging people.

Osteoporosis is a silent disease without obvious symptom and evidence until occurrence of fracture. Early diagnosis of osteoporosis is the key issue for efficient treatment and for identification of osteoporotic patient with high risk of fracture. At present, diagnosis of osteoporosis and assessment of fracture risk are based on the quantitative analysis of BMD by DXA. However, the gold standard method of BMD assessment of bone mass by DXA only partially provides the information about bone strength. The osteoporosis is characterized by bone fragility. Bone fragility is evaluated by its microarchitectural quality which is identified by all bone features such as microarchitecture, microdamage and remodeling rates with the influence of bone’s ability for resistant fracture. The two-dimensional technique of DXA reveals the intrinsic limitations to discriminate cortical from cancellous bone and characterize changes because of bone geometry. Recently, several approaches have been developed to provide supplementary information for the assessment of fracture risks except BMD. For example, quantitative computed tomography (QCT) has been developed to evaluate bone loss [4, 5]. In QCT, the true value of mineral density for trabecular bone has been obtained from cortical portion of bone [6]. Moreover, the instrument of ultrasound has been applied to examine bone condition and fracture risk [7, 8]. The novel ultrasonic instrument is the first tool to characterize bone and assess microarchitecture with the detection of central axial reference sites (lumbar vertebrae and proximal femur) without the uses of ionizing radiations. However, these imaging techniques still have some limitations such as bulky device, high cost and limited accessibility to impede their applications for widespread uses and primary care inspection. Besides imaging techniques, the alternative method is still essential to be developed for the early and correct diagnosis of osteoporosis because the accurate diagnosis of osteoporosis in the early stage leads to a better management for the prevention and treatment.

**Fig. 1:**
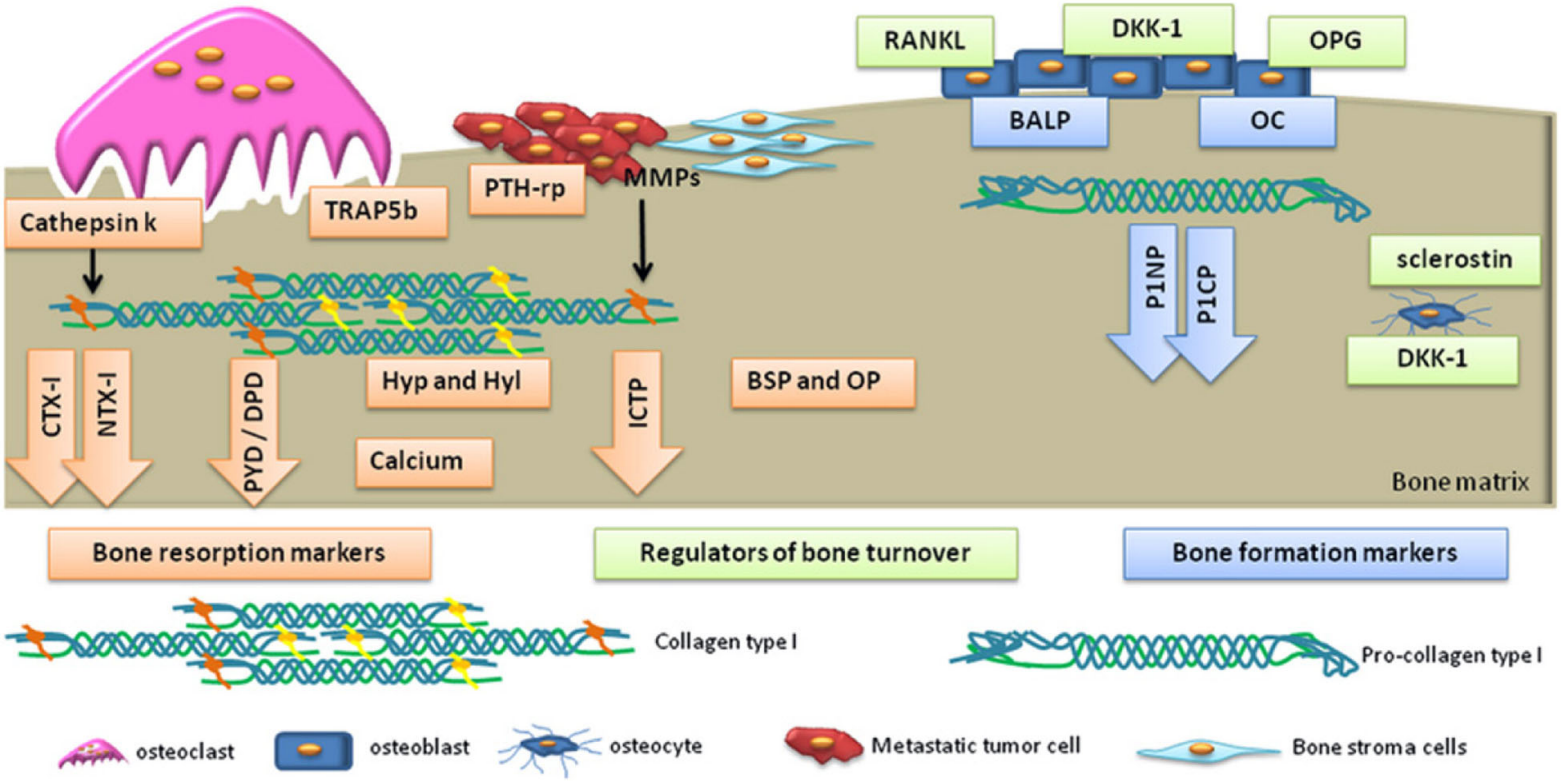
Biochemical biomarkers of bone turnover. *Blue* boxes/arrows represent bone formation markers: bone-specific alkaline phosphatase (BALP); osteocalcin (OC); propeptides of type I procollagen (P1NP and P1CP). Orange boxes/arrows represent bone resorption markers: pyridinoline (PYD); deoxypyridoline (DPD); carboxy-terminal crosslinked telopeptide of type 1 collagen (CTX-1); amino-terminal crosslinked telopeptide of type 1 collagen (NTX-1); hydroxyproline (HYP); hydroxylysine (HYL); bone sialoprotein (BSP); osteopontin (OP); tartrate-resistant acid phosphatase 5b (TRAP 5b); cathepsin K (CTSK). *Green* boxes represent regulators of bone turnover: receptor activator of NF-κB ligand (RANKL), osteoprotegerin (OPG), dickkopf-1 (DDK-1) and sclerostin. Reproduced with permission from Ref. [9]. Copyright @ 2015, Nature Publishing Group

The biomarkers of bone turnover have been investigated in the past decade. As shown in Fig. 1, the mechanism of bone remodeling is composed by bone resorption and bone formation [9]. Bone biomarkers are produced from the bone remodeling process included bone formation biomarkers, bone resorption biomarkers and regulators of bone turnover. Detections of bone metabolism have been studied with the biomarkers of enzymes, proteins and by-products during the bone remodeling process [10–13]. Various biomarkers are now available for specific and sensitive assessment of the rate for bone formation and bone resorption as shown in Table 1 [14]. For example, the bone formation biomarkers are total alkaline phosphatase (ALP), bone-specific alkaline phosphatase (BALP), osteocalcin (OC), procollagen type 1 N-terminal propeptide (P1NP) and procollagen type 1 C-terminal propeptide (P1CP). The bone resorption biomarkers are hydroxyproline (HYP), hydroxylysine (HYL), deoxypyridinoline (DPD), pyridinoline (PYD), bone sialoprotein (BSP), osteopontin (OP), tartrate-resistant acid phosphatase 5b (TRAP 5b), carboxy-terminal crosslinked telopeptide of type 1 collagen (CTX-1), amino-terminal crosslinked telopeptide of type 1 collagen (NTX-1) and cathepsin K (CTSK). The regulators of bone turnover are receptor activator of NF-kB ligand (RANKL), osteoprotegerin (OPG), dickkopf-1 (DDK-1) and sclerostin. These biomarkers are useful to provide the early assessment of osteoporosis when the BMD measurement of DXA does not offer enough information to make the diagnosis. Therefore, the combination of BMD measurement by DXA and bone biomarker detections shows the great potential to improve the early assessment of people with the high risk of osteoporosis. Herein, we present the recent studies and breakthroughs in the detections of bone related biomarkers for the assessment of osteoporosis. We hope to provide a comprehensive mini-review starting from the functions of bone biomarkers included bone formation, bone resorption and regulator of bone turnover to the applications in osteoporotic assessment at the end.

**Table 1:** Summary of detection techniques for bone biomarkers

Biomarker	Method	Concentration	Reference
Total ALP	standard Technicon Auto-analyzer	113 U/L	[12]
Total ALP	Roche COBAS Integra 800	>129 U/L	[13]
Total ALP	Olympus AU 5200 analyzer	64.8–79.7 U/L	[14]
BALP	enzyme immunoassay	24.9–19.7 U/L	[24]
BALP	enzyme immunoassay	66.4 ± 8.7 U/L	[25]
OC	antibody immunoassay	4.1 ± 0.5 ng/mL	[27]
OC	ELISA	16.16 ± 4.5 ng/mL	[28]
P1NP	Elecsys 2010 automated analyzer	54.1 μg/L	[29]
P1CP	radioimmunoassay	97–116 ng/mL	[32]
HYP	Bergman and Loxley method	34.7 mg/g creatinine	[35]
GHYL	HPLC	1.35 ± 0.82 mmol/mol	[39]
GHYL	HPLC	1.93–6.07 μmol/L	[40]
DPD	HPLC	11.3–22.3 nmol/L	[40]
DPD	chemiluminescence immunoassay	4.7 nmol/L	[42]
DPD	enzyme immunoassay	4.4 nmol/L	[42]
PYD	HPLC	28.8 μmol/mol creatinine	[45]
BSP	radioimmunoassay	12.1 ± 5.0 μg/L	[48]
BSP	radioimmunoassay	8.0 μg/L	[49]
OP	ELISA	20.75 ± 5.36 ng/mL	[51]
TRAP 5b	enzyme immunoassay	4.0 U/L	[54]
TRAP 5b	enzyme immunoassay	3.40 ± 0.87 U/L	[55]
CTX-1	ELISA	0.17–0.30 ng/ml	[56]
NTX-1	ELISA	37 ± 15 nmol BCE/mmol Cr	[57]
CTSK	ELISA	10.17 pmol/L	[59]
RANKL	ELISA	0.08 pmol/L	[61]
OPG	ELISA	1.8 pmol/L	[61]
DDK-1	ELISA	34.3 pmol/L	[65]
Sclerostin	ELISA	29.5 pmol/L	[65]

## Bone formation biomarkers

### Total alkaline Phosphatase (ALP)

Alkaline phosphatase is an enzyme found in the bloodstream. The different forms of ALPs can break down proteins in the human body. Most of ALPs are produced in liver and some of ALPs are generated in the bones, intestines and kidneys. The total ALP is examined by the measurement of the amount of alkaline phosphatase enzyme in the bloodstream. The measurement of total ALP only needs a simple blood draw and is often a routine part of blood tests. The normal levels of ALP with people are various by age, blood type, gender and pregnancy. Unusual concentrations of ALP in blood generally indicate an issue with liver, gall bladder, or bones. The ALP test can be used for diagnosis of bone problems such as rickets, osteomalacia and Paget’s disease. The ALP level also can be applied to check the treatment efficiency for the above conditions. For example, the standard Technicon Autoanalyzer has been used to measure the serum ALP for osteoporotic patients with inflammatory bowel disease [15]. The spinal bone mineral content has revealed significant correlations with serum total ALP. Recent study has also shown that the activity of serum total ALP >129 U/L is used as an indicator for osteoporosis in men [16]. Moreover, the decrease of total ALP has been demonstrated with the treatment with alendronate from 79.7 U/L to 64.8 U/L [17]. The results indicate that total ALP can be used as an indicator to reflect the efficiency of drug treatment for osteoporosis. However, small change and wide extensive range of total ALP may be led to wrong diagnosis of osteoporosis for postmenopausal women.

### Bone-specific alkaline Phosphatase (BALP)

With normal liver function in adults, about 50% of total ALP is produced from bone in serum. The ALP isoenzymes produced from bone are difficult to distinguish with the isoenzymes from liver because they are only different by posttranslational glycosylation. Several approaches have been applied to specific measurements of BALP such as agarose electrophoresis, heat and chemical inactivation, wheat germ agglutinin precipitation, wheat germ agglutinin-high performance liquid chromatography, immunoradiometric assay and enzyme immunoassay [18–26].

For the enzyme immunoassay of BALP, the detection limit is 0.7 U/L and the mean values are 24.9 ± 7.0 U/L and 19.7 ± 5.6 U/L for men and premenopausal women, respectively. [27] With osteoporosis, the BALP activity of 66.4 ± 8.7 U/L has been detected with women aged over 59 years [28]. As an indicator of osteoblastic activity, the measurement of BALP is applied as the assistance for the management of osteoporosis in premenopausal and postmenopausal women.

### Osteocalcin (OC)

Osteocalcin, also known as bone gamma-carboxyglutamic acid-containing protein, is a small protein composed by 49 amino acids. OC is synthesized by mature osteoblasts, odontoblasts and hypertrophic chondrocytes. Moreover, OC is the most abundant non-collagenous protein in bone comprised about 2% of total protein in the human body. OC produced by osteoblasts plays an important role for metabolic regulation, bone mineralization and calcium ion homeostasis [29]. OC has been demonstrated that the level of serum OC is highly correlated with the increase of BMD during treatment with bone formation drugs for osteoporosis. Serum OC has been considered a specific biomarker of osteoblast function for evaluation of bone formation rate in osteoporosis. In many studies, osteocalcin has been demonstrated as an important biomarker to investigate the efficiency for the drug on bone formation. For instance, with the RNAse-enriched-LF supplementation, the change of OC from baseline (7.2 ± 2.8%) has shown a promising and favorable effect in postmenopausal women [30]. Furthermore, the mean levels of osteocalcin have revealed a significant difference between the postmenopausal osteoporotic (16.16 ± 4.5 ng/ml) and non-osteoporotic (11.26 ± 3.07 ng/ml) women [31]. The uses of osteocalcin as a bone formation biomarker could provide the advantages such as tissue specificity, wide availability and low variation. The bone remodeling biomarker of serum OC may be useful for the assessment of osteoporosis and for the prediction of the fracture risk in elderly persons, especially in women.

### Procollagen type 1 N-terminal Propeptide (P1NP)

Type 1 collagen can be found in the organic bone matrix (> 90%), which is developed in bone from procollagen type 1. Procollagen type 1 is synthesized by fibroblasts and osteoblasts. Procollagen type 1 has N-terminal and C-terminal extensions, which are removed by specific proteases during conversion of procollagen to collagen. The procollagen type 1 included P1CP and P1NP are subsequently conjugated onto the bone matrix. The bone formation biomarker of P1NP is a specific indicator of type 1 collagen deposition. P1NP is released during the formation of type 1 collagen into the intracellular space and P1NP eventually exists in the blood stream. P1NP is usually released in the trimeric structure (derived from the trimeric collagen structure) and then is rapidly broken down to a monomeric form by thermal degradation effects. Antibodies of P1NP are used to detect the trimeric structure of P1NP by enzyme-linked immunosorbent assay (ELISA) or radioimmunoassay. Recently, for the postmenopausal women with osteoporosis participating in the Parathyroid Hormone and Alendronate for Osteoporosis study, the mean value (54.1 μg/L) of total P1NP before initiating therapy is 74% higher than that in healthy premenopausal women with the age above 35 [32]. P1NP has been demonstrated to be a more sensitive bone biomarker to measure the bone formation rate in osteoporosis. The measurement of P1NP is being developed for clinical application.

### Procollagen type 1 C-terminal Propeptide (P1CP).

P1CP is a single protein with molecular weight of 115 kDa containing mannose-rich carbohydrate side chains inserted post-translationally. PICP is cleared by liver endothelial cells via the mannose receptor and therefore has a short serum half-life of 6–8 min [33, 34]. Recent study shows that the P1CP in serum is used as a biomarker of bone formation to evaluate the effect of nandrolone decanoate and female sex hormones. The mean initial PICP concentration in the women with a vertebral fracture (97 ± 23 ng/mL) is significantly lower than that in the women with a forearm fracture (116 ± 27 ng/mL) [35]. Currently, both of P1NP and P1CP are analyzed by specific immunoassays. However, for the analyses of procollagen type 1, the biomarker of P1NP is more extensively investigated in the literature than that of P1CP. Several studies have also demonstrated good correlations between serum P1CP levels and the rate of bone formation [36].

## Bone Resorption biomarkers

### Hydroxyproline (HYP)

HYP is an amino acid derived from the post-translational hydroxylation of proline. HYP provides about 12–14% of the total amino acid content of mature collagen. During the degradation of bone collagen, about 90% of the HYP is released and then the HYP is primarily metabolized in the liver [37]. The level of HYP has significantly increased in urine with postmenopausal osteoporosis women in comparison with the postmenopausal nonosteoporosis women [38]. The increase of urinary HYP indicates that the degradation of collagen type I from the bone matrix is raised in osteoporotic women. Although the HYP is principally used as a resorption biomarker, about 10% of the HYP is derived from newly synthesized procollagen peptides during bone formation. Moreover, HYP can be found in other tissues such as skin and cartilage and also can be liberated from the metabolism of elastin and C1Q [39]. HYP is consequently considered as a non-specific bone resorption biomarker of collagen turnover. The use of HYP has been replaced by more sensitive and specific biomarkers for the assessment of osteoporosis [40].

### Hydroxylysine (HYL)

HYL is an amino acid derived from a post-translational hydroxy modification of lysine. HYL has two forms included galactosyl hydroxylysine (GHYL) and glucosyl-galactosyl-hydroxylysine (GGHYL) [41]. GHYL and GGHYL are both released into the circulation during collagen degradation. GHYL is a more specific for bone biomarker because it is only derived from bone resorption. On the other hand, GGHYL can be found in skin and complement molecule of C1Q. Therefore, GHYL is considered as a better bone resorption biomarker than GGHYL and HYP. In previous work, the concentration of GHYL in urinary excretion has been applied to evaluate the occurrence of fracture in postmenopausal osteoporotic women without fragility fractures and postmenopausal osteoporotic women with fragility fractures [42]. The high levels of GHYL in fracture patients suggest a possible defect in bone collagen and the urinary GHYL may indeed identify such an abnormality. Furthermore, GHYL can be easily measured without preanalytical hydrolysis and extraction by high performance liquid chromatography (HPLC) [43]. Although the GHYL has revealed the potential as a biomarker of bone resorption, the use of GHYL has not been extensively studied for the assessment of osteoporosis and the evaluation of osteoporotic treatment. The clinical application of GHYL is still limited because of the absence of facile routine methods for its measurement.

### Deoxypyridinoline (DPD)

DPD is a molecule to mechanically stabilize collagen by crosslinking between individual collagen peptides [44]. During the process of bone resorption, the crosslinked collagens are proteolytically broken down and then the DPD is released into the circulation and excreted by urine. Most of DPD are found in the bone and dentin. Therefore, DPD is used as a specific biomarker for bone resorption. In previous work, the DPD has been pretreated with preanalytical hydrolysis and extraction before HPLC analysis because DPD is excreted in the urine in free (40%) and peptide-bound (60%) forms. To improve the accuracy, the peptide-bound form is transferred into free form for the HPLC measurement. The drawbacks for HPLC measurement of DPD are complicated procedure and variable recovery [43]. Recently, the automated chemiluminescence immunoassay and enzyme immunoassay have been developed for the direct detection of urinary free DPD. The experimental results of chemiluminescence immunoassay and enzyme immunoassay methods have shown the correlation with HPLC measurement of urinary free DPD [45–47]. The measurements of free DPD in urine by the immunoassay approaches have provided the possibility for the clinical application in the monitoring of patients with bone pathology and metabolic bone disease.

### Pyridinoline (PYD)

Collagen crosslink of PYD is produced during the extracellular maturation of fibrillar collagens and released into the circulation from degradation of mature collagens. Previous study shows that the PYD exhibits long-term chemical stability in both the free and conjugated forms by HPLC analyses [48]. However, PYD is found in cartilage, bone, ligaments and blood vessels. Therefore, PYD is a non-specific bone resorption biomarker in comparison with DPD.

### Bone Sialoprotein (BSP)

BSP is a phosphorylated glycoprotein with an apparent molecular weight of 60–80 kDa [49]. BSP is an element of mineralized tissues such as bone, dentin, cementum and calcified cartilage. BSP is an important component of the bone extracellular matrix and has been demonstrated to for the formation of approximately 8% of all non-collagenous proteins found in bone and cementum [50]. BSP is generated by osteoblasts, odontoblasts and osteoclasts. Therefore, BSP is considered as an important factor for cell-matrix adhesion processes and stimulation of osteoclast-mediated bone resorption. Many studies have developed immunoassays for the measurement of BSP in serum. For example, radioimmunoassay measurements have obtained mean serum BSP concentrations of 12.1 ± 5.0 μg/L from 90 healthy controls and 32.3 ± 17.3 μg/L from patients with Paget disease [51]. Serum levels of BSP have been also demonstrated the significant correlation with BALP and OC in osteoporosis patients [52]. BSP has shown great potential as a bone resorption biomarker for osteoporotic assessment.

### Osteopontin (OP)

OP is a phosphorylated glycoprotein expressed by transformed cells, macrophages, activated T-lymphocytes, specialized epithelial cells and bone cells [53]. Recent study shows that women with OP over-expression reveal less resistance to postmenopausal osteoporosis than women with normal OP levels [54]. The levels of plasma OP could be used as a biomarker to evaluate the treatment of intermittent parathyroid hormone in menopausal osteoporosis.

### Tartrate-resistant acid Phosphatase 5b (TRAP 5b)

TRAP 5b is normally secreted by osteoclasts during bone resorption [55, 56]. Thus, TRAP 5b is used as a reference for osteoclast activity and numbers. In the circulation, the hydrolyzed TRAP 5b is metabolized in the liver and then excreted in the urine. TRAP 5b can be specifically detected in serum by immunoassays. Previous report shows that serum TRAP 5b has been used to identify limited or extensive bone metastasis in breast cancer patients [57]. Furthermore, serum TRAP 5b has been applied to monitor the efficiency of alendronate treatment [58]. The bone resorption biomarker of TRAP 5b is extensively studied and revealed good specificity and high sensitivity in comparison with other bone biomarkers.

### Carboxy-terminal Crosslinked Telopeptide of type 1 collagen (CTX-1),

Telopeptides of type 1 collagen are extensively investigated and used bone resorption biomarkers included carboxy-terminal crosslinked (CTX-1) and amino-terminal crosslinked (NTX-1). CTX-1 and NTX-1 are both are released during collagen degradation. ELISA is used to measured CTX-1 with a monoclonal antibody against an octapeptide sequence (EKAHD-β-GGR) in the α-1 (I) chain of the β-isoform. Recent study has shown that CTX-1 is a specific and sensitive biomarker of bone resorption that can rapidly indicate the response to bisphosphonate therapy for postmenopausal osteoporosis [59]. However, serum CTX-1 is influenced by food intake and blood withdrawal must take place in the fasting state because food intake substantially decreases the levels of CTX-1 [60].

### Amino-terminal Crosslinked Telopeptide of type 1 collagen (NTX-1)

NTX-1 is stable in urine at room temperature for up to 24 h and is usually quantified by ELISA with urine sample. The urinary NTX-1 has been used as a bone resorption biomarker to assess the risk of fracture in postmenopausal women [60]. The urinary NTX-1 is selected as the preferred biomarker compared with serum CTX-1 for practical application because it is not affected by food intake and it prevents blood withdrawal.

### Cathepsin K (CTSK)

Cathepsins are members of the cysteine protease family with 11 isoforms. CTSK is defined by its high specificity for kinins. CTSK is mainly expressed at the ruffled border of actively resorbing osteoclasts [61]. Osteoclasts secrete CTSK into bone resorption defect for degradation of bone matrix proteins included type 1 collagen, osteopontin and osteonectin. Therefore, CTSK is an important factor in process of bone resorption. The level of CTSK has revealed significantly different between controls and patients with osteoporosis [62]. The result indicates that serum level of CTSK could serve as a potential biomarker for fracture prediction and bone-mineral density.

## Regulators of bone turnover

### Receptor activator of NF-κB ligand (RANKL)

During the process of bone remodeling, osteoblasts produce RANKL and OPG to regulate the differentiation and maturation of osteoclasts. Recently, dextromethorphan has been demonstrated the inhibition of RANKL-induced osteoclastogenesis and bone resorption by abrogating the activation of NF-κB signaling in vitro. The oral administration of dextromethorphan improves ovariectomy-induced osteoporosis in vivo [63]. Serum levels of RANKL from humans have been observed for assessments of the states in metabolic bone diseases [64]. Although the serum RANKL has been studied for fracture risk prediction and evaluation of the response from osteoporosis treatment, many works still need to be investigated for the clinical application of RANKL.

### Osteoprotegerin (OPG)

OPG is generally considered to be a secreted soluble receptor and is produced by many different tissues and cell types including osteoblasts. The role of OPG is used as a decoy receptor for RANKL and inhibitor of osteoclastogenesis [65]. Studies in mice have revealed that the OPG knockout mouse develops severe osteoporosis, whereas the overexpression of OPG in transgenic mouse models and OPG treatment of normal mice leads to osteopetrosis [66]. OPG can be measured in serum, plasma EDTA, citrate and heparin samples. There are commercially available sandwich ELISA assays for analyzing OPG by using a monoclonal capture and polyclonal detection antibodies [64]. However, the clinical use of serum OPG as a biomarker for evaluation of bone disease activity still needs additional demonstration.

### Dickkopf-1 (DDK-1)

DKK-1 and sclerostin are the inhibitors of Wnt signaling and are applied as bone remodeling biomarkers. DKK-1 is produced by osteoblasts and is secreted into circulation. The serum levels of DDK-1 reflect the inhibition of bone formation [67]. DKK-1 levels have decreased from 34.3 pmol/L at baseline to 29.7 pmol/L at the 24-month of the breast cancer patients with anastrozole treatment [68]. DKK-1 has shown the correlation with the BMD of the femoral neck and of the total hip. Further long-term studies are necessary to identify the clinical application of the regulator DKK-1 as a biomarker for assessment of osteoporosis.

## Sclerostin

In the presence of sclerostin, the Wnt pathway is downregulated and consequently osteoblastic differentiation is inhibited. Sclerostin is produced by osteocytes. Sclerostin is secreted into circulation, and serum levels reflect inhibition of bone formation [67]. In previous work, the concentrations of serum sclerostin have significantly increased from 29.5 pmol/L at baseline to 43.2 pmol/L after 24 months of treatment with anastrozole in breast cancer patients [68]. However, the clinical trial is further needed for the use of sclerostin as a biomarker of bone turnover.

## Conclusions

In this mini-review, we reviewed several investigations and developments for the applications of bone biomarkers in the assessments of bone related diseases. From laboratory tests to clinical trials, the possibility of bone biomarkers to evaluate bone remodeling processes has been extensively demonstrated in physiological and pathological states. Bone biomarkers have shown great potential to serve as powerful indicators to evaluate the osteoporosis therapy and even to assist the clinical diagnosis of osteoporosis in the early stage. **Among these bone biomarkers, P1NP has shown the great potential as a sensitive and stable bone biomarker for the early detection of osteoporosis.**

## Abbreviations

ALP: Total alkaline phosphatase
BALP: Bone-specific alkaline phosphatase
BMD: Bone mineral density
BSP: Bone sialoprotein
CTSK: Cathepsin K
CTX-1: Carboxy-terminal crosslinked telopeptide of type 1 collagen
DDK-1: dickkopf-1
DPD: Deoxypyridinoline
DXA: Dual-energy X-ray absorptiometry
HYL: Hydroxylysine
HYP: Hydroxyproline
NTX-1: Amino-terminal crosslinked telopeptide of type 1 collagen
OC: Osteocalcin
OP: Osteopontin
OPG: Osteoprotegerin
P1CP: Procollagen type 1 C-terminal propeptide
P1NP: Procollagen type 1 N-terminal propeptide
PYD: Pyridinoline
QCT: Quantitative computed tomography
RANKL: Receptor activator of NF-kB ligand
TRAP 5b: Tartrate-resistant acid phosphatase 5b (TRAP 5b)
WHO: World Health Organization
